# An Educational Curriculum for Residents, Advanced Practice Providers, and Fellows in Cardiac Intensive Care Units

**DOI:** 10.1016/j.jacadv.2025.102110

**Published:** 2025-08-28

**Authors:** Anthony P. Carnicelli, Balimkiz C. Senman, P Elliott Miller, Garima Dahiya, Jacob C. Jentzer, Manoj S. Ambalavanan, Amanda C. Garfinkel, Aimee Zaas, Elizabeth Poindexter, Dan P. Judge, Shashank S. Sinha, David D. Berg, Andrea M. Elliott, David A. Morrow, Jason N. Katz

**Affiliations:** a Division of Cardiology, Department of Medicine, Medical University of South Carolina, Charleston, South Carolina, USA; b Division of Cardiology, Department of Medicine, Duke University, Durham, North Carolina, USA; c Section of Cardiovascular Medicine, Yale School of Medicine, New Haven, Connecticut, USA; d Division of Critical Care Cardiology, Department of Cardiovascular Medicine, Mayo Clinic, Rochester, Minnesota, USA; e Division of Cardiology, Department of Medicine, Brigham and Women’s Hospital, Boston, Massachusetts, USA; f Division of Infectious Disease sand International Health, Department of Medicine, Duke University, Durham, North Carolina, USA; g Division of Pulmonary and Critical Care Medicine, Department of Medicine, Medical University of South Carolina, Charleston, South Carolina, USA; h Inova Schar Heart and Vascular, Inova Fairfax Medical Campus, Falls Church, Virginia, USA; i Division of Cardiology, Department of Medicine, University of Minnesota, Minneapolis, Minnesota, USA; j Leon H Charney Division of Cardiology, New York University Grossman School of Medicine & Bellevue Hospital, New York, New York, USA

**Keywords:** cardiac intensive care unit, CICU, critical care cardiology, medical education

## Abstract

The contemporary cardiac intensive care unit (CICU) serves as a dynamic educational environment for postgraduate physicians and advanced practice provider trainees. This educational experience, however, can vary substantially between institutions. Specific learning objectives are needed to standardize the educational experience for trainees rotating through the contemporary CICU. We provide a structured, CICU-based curriculum emphasizing exposure to a wide spectrum of cardiovascular pathologies and incorporating learner progression from early to advanced stages, adaptable to a variety of training pathways. Prioritizing standardized educational objectives during training will better prepare learners for further subspecialty training programs and the complexities of modern CICU-based practice.

## Highlights

• Core clinical competencies should be established for residents, advanced practice providers, and cardiology fellows rotating through the CICU.
• We propose a core educational curriculum that may be used by training program directors and CICU directors to standardize CICU-based education.
• Future efforts to investigate the relative efficacy of various educational modalities in a busy CICU practice environment are warranted.

The contemporary cardiac intensive care unit (CICU) offers a rich educational environment in which postgraduate physician trainees in internal medicine, cardiology, and other subspecialties rotate. Postgraduate critical care (CC) fellowship programs for advanced practice providers also incorporate CICU rotations as a key component of training.^1^ Broad training recommendations for resident and fellow physicians are outlined in the Accreditation Council for Graduate Medical Education^2^^,^^3^ and Core Cardiology Training Statement^4^ documents. These documents, however, do not describe specific clinical competencies that trainees should learn during CICU rotations. Similarly, no specific cardiovascular critical care (CVCC) competencies are defined for advanced practice provider CC fellows. We aimed to develop a core educational curriculum for trainees rotating in the CICU, providing a resource for CICU directors, training program directors, and clinician educators who seek to enhance the CICU educational experience.

CVCC encompasses a broad spectrum of interrelated cardiac and noncardiac conditions with an expanding array of medical and procedural treatment options.^5^ Accreditation Council for Graduate Medical Education provides no specific requirements for CICU training during cardiovascular disease fellowship. Core Cardiology Training Statement recommends a minimum of 8 weeks of CICU training during the first 24 months of cardiovascular disease fellowship for level 1 competency.^4^^,^^6^ The primary objectives of these CICU rotations are to:

1) gain exposure to a diverse spectrum of cardiovascular conditions leading to critical illness,
2) develop proficiency in diagnosing and managing these conditions,
3) gain experience managing noncardiac critical illness in the setting of concomitant cardiovascular disease, and
4) apply a collaborative, multidisciplinary, team-based approach to CVCC.

While this document focuses primarily on cardiovascular pathologies, diagnosis and management of noncardiac pathologies in the setting of concomitant cardiovascular disease is a crucial component of a CICU educational curriculum. Similarly, CC procedural competencies (eg, central venous catheterization, arterial line placement, and endotracheal intubation) are essential skills that should be developed during CICU and noncardiac intensive care unit rotations. This document specifically addresses the cardiovascular procedural competencies most relevant to the CICU patient population, including pericardiocentesis, transvenous pacemaker placement, and pulmonary artery catheterization.

The Central Illustration outlines the core clinical topics that should be covered as trainees progress through their CICU rotations, recognizing that not all topics will require the same depth of discussion. Educators and learners should incorporate primary research and guideline recommendations in the educational experience to encourage an understanding of the strengths and limitations of the evidence base for each topic. CICU-focused education typically involves learners with varying degrees of experience, making it necessary to teach across a broad educational spectrum. For each topic, skills are categorized along a continuum from “early” to “advanced learner.” While this framework provides a conceptual structure, learning in each domain will likely not be acquired in a linear fashion. The number and duration of CICU rotations vary between institutions and even within a single institution across training programs. Typically, these rotations are 1 to 4 weeks in duration, incorporating both daytime and nighttime work hours, with varying durations of consecutive work hours. For trainees with limited CICU exposure, achieving advanced learner status in all or even most clinical topics may not be feasible. In such cases, CICU educators may choose to review this educational curriculum with the learner in advance and tailor the learner’s experience to specific goals.

**Central Illustration:**
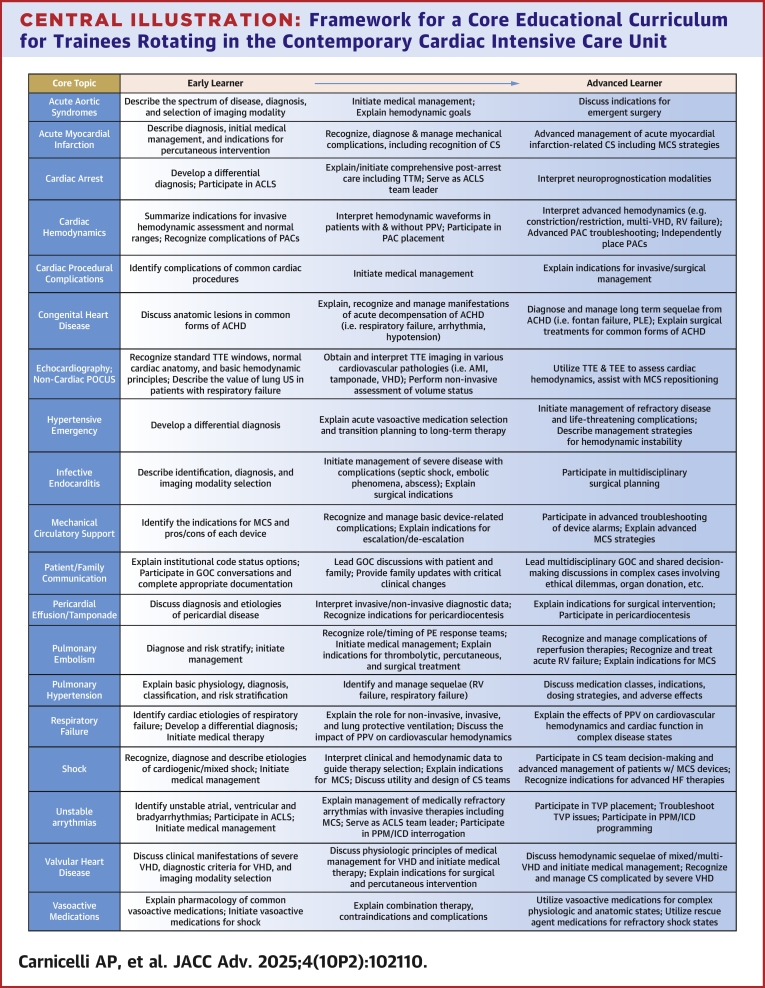
**Framework for a Core Educational Curriculum for Trainees Rotating in the Contemporary Cardiac Intensive Care Unit** ACHD = adult congenital heart disease; ACLS = advanced cardiovascular life support; AMI = acute myocardial infarction; CS = cardiogenic shock; GOC = goals of care; ICD = implantable cardioverter defibrillator; MCS = mechanical circulatory support; PAC = pulmonary artery catheter; PE = pulmonary embolism; PLE = protein-losing enteropathy; POCUS = point of care ultrasound; PPM = permanent pacemaker; PPV = positive pressure ventilation; RV = right ventricle; TEE = transesophageal echocardiography; TTE = transthoracic echocardiography; TTM = targeted temperature management; TVP = transvenous pacemaker; US = ultrasound; VHD = valvular heart disease

Guided by adult learning principles, a variety of educational strategies should be employed to optimize learning.^7^^,^^8^ Two of the most widely used formats include structured didactic sessions and bedside teaching. While most of the clinical topics discussed here are well-suited to both formats, incorporating alternative teaching strategies can reinforce key concepts and enhance learner engagement. Suggested alternatives include topic-focused journal clubs, case-based learning, simulation-based sessions, and problem-based learning.^9^^,^^10^ Teaching is often best received when it occurs “organically” rather than being templated to occur at predetermined times, particularly when it applies to a currently admitted patient. Redundancy and temporally spaced repetition for more advanced learners play crucial roles in knowledge retention. Structured didactic sessions may include in-person teaching or prerecorded online modules for asynchronous teaching, with a comprehensive structured curriculum covering the gamut of topics in CVCC. Simulation-based education may be particularly effective in enhancing communication skills, improving interprofessional team performance, and maintaining competency in high-acuity, low-frequency scenarios and procedures.^11^ Importantly, education must be a priority for the entire team, rather than something that is reserved for clinical downtime—which, in a high-acuity setting, may never come. Providing an array of teaching opportunities will help to engage different styles of learners.

Active engagement from all members of a team is essential to success. While leveraging team-member expertise is valuable, trainees and educators should be encouraged to explore areas where they feel less comfortable, as the ability to teach a topic is well recognized as a necessity on the journey toward mastery. Although the attending cardiologist’s presence during educational efforts is vital, trainees at all levels should take an active role in leading educational sessions, as each member of the multidisciplinary CICU team contributes a unique and valuable perspective. CICU-based educators should also encourage broad participation in educational initiatives, inviting nurses, physical therapists, nutritionists, pharmacists, cardiogenic shock coordinators, and other key team members to participate in and lead educational sessions.

Several factors may limit the generalizability of this framework. Clinical practice patterns and patient populations vary significantly between institutions, requiring adaptation to align with the capabilities of an individual training program. Many institutions employ standardized management algorithms for complex conditions such as cardiogenic shock and cardiac arrest. These protocols should be incorporated into institution-specific training whenever possible. Lastly, trainees must be expected to dedicate time to focused, self-guided learning outside of the clinical setting to aid in progress toward becoming an advanced learner.

A robust educational experience for trainees is a cornerstone of contemporary CICU practice. The core topics outlined here provide a framework, offering trainees both breadth and depth across the most common cardiovascular pathologies encountered in the CICU. By prioritizing education and fostering engagement from all members of the clinical team, institutions can create a comprehensive CVCC learning environment.

## Funding support and author disclosures

Dr Carnicelli has received research support from Acorai and 10.13039/100020297Abiomed paid to the institution and speaker honoraria and travel support from 10.13039/100020297Abiomed. Dr Elliott has received an educational honorarium from 10.13039/100015345Zoll. Dr Katz has received speaker honoraria from 10.13039/100020297Abiomed and 10.13039/100015345Zoll and nonfinancial research support from 10.13039/100000046Abbott. All other authors have reported that they have no relationships relevant to the contents of this paper to disclose.
